# Exploring the Potential of Three-Dimensional Imaging, Printing, and Modeling in Pediatric Surgical Oncology: A New Era of Precision Surgery

**DOI:** 10.3390/children10050832

**Published:** 2023-05-03

**Authors:** Arnau Valls-Esteve, Núria Adell-Gómez, Albert Pasten, Ignasi Barber, Josep Munuera, Lucas Krauel

**Affiliations:** 1 Innovation Department, SJD Barcelona Children’s Hospital, Santa Rosa 39-57, 08950 Esplugues de Llobregat, Spain; 2 Medicina i Recerca Translacional, Facultat de Medicina i Ciències de la Salut, Universitat de Barcelona, 08036 Barcelona, Spain; 3 3D for Health Unit (3D4H), SJD Barcelona Children’s Hospital, Universitat de Barcelona, 08950 Esplugues de Llobregat, Spain; 4 Pediatric Surgical Oncology Unit, Department of Pediatric Surgery, SJD Barcelona Children’s Hospital, Universitat de Barcelona, 08950 Esplugues de Llobregat, Spain; 5 Department of Diagnostic Imaging, SJD Barcelona Children’s Hospital, Universitat de Barcelona, 08950 Esplugues de Llobregat, Spain

**Keywords:** personalized medicine, three-dimensional printing, virtual reality, pediatrics, oncology, surgery, children, model

## Abstract

Pediatric surgical oncology is a technically challenging field that relies on CT and MRI as the primary imaging tools for surgical planning. However, recent advances in 3D reconstructions, including Cinematic Rendering, Volume Rendering, 3D modeling, Virtual Reality, Augmented Reality, and 3D printing, are increasingly being used to plan complex cases bringing new insights into pediatric tumors to guide therapeutic decisions and prognosis in different pediatric surgical oncology areas and locations including thoracic, brain, urology, and abdominal surgery. Despite this, challenges to their adoption remain, especially in soft tissue-based specialties such as pediatric surgical oncology. This work explores the main innovative imaging reconstruction techniques, 3D modeling technologies (CAD, VR, AR), and 3D printing applications through the analysis of three real cases of the most common and surgically challenging pediatric tumors: abdominal neuroblastoma, thoracic inlet neuroblastoma, and a bilateral Wilms tumor candidate for nephron-sparing surgery. The results demonstrate that these new imaging and modeling techniques offer a promising alternative for planning complex pediatric oncological cases. A comprehensive analysis of the advantages and limitations of each technique has been carried out to assist in choosing the optimal approach.

## 1. Introduction

Pediatric surgical oncology represents a complex field of pediatric surgery, requiring mastery of all its technical aspects, including thoracic, brain, urology, and abdominal surgery. 

In recent years, advances in imaging and interventional technology, such as three-dimensional imaging, minimally invasive surgery, fluorescence-guided surgery, and robotic-assisted surgery, have shown a promising improvement in safety and precision, and a potential improvement in clinical outcomes [1,2,3,4,5]. However, all of these technologies are still not standardized in clinical practice and they still encounter challenges to their wider adoption, such as their high cost and a lack of reimbursement. Other innovations such as 3D modeling and 3D printing [6,7,8,9] have increased their adoption in the surgical field exponentially in recent years, becoming especially popular in bone surgical specialties due to the ease of bone segmentation (3D virtual reconstruction of the images). Unfortunately, this is not the case for other soft-tissue-based specialties, as in the case of pediatric surgical oncology [8,10], in which segmentations must be performed manually or semi-automatically.

The relatively low volume of pediatric oncology patients compared to the adult surgical oncology population makes it difficult to develop clinical research and innovation in pediatric surgical oncology. Hence, there is a lack of technological innovations specifically designed and developed for this field in comparison to other adult or bone-based surgical specialties such as maxillofacial surgery, cardiac surgery, and neurosurgery [1,11]. However, the high complexity of pediatric oncology and the nature of the patients involved (in constant growth and anatomical evolution) demands highly personalized solutions and surgical tools to help improve the already very high skill level needed for surgeons in this field.

**Figure 1:**
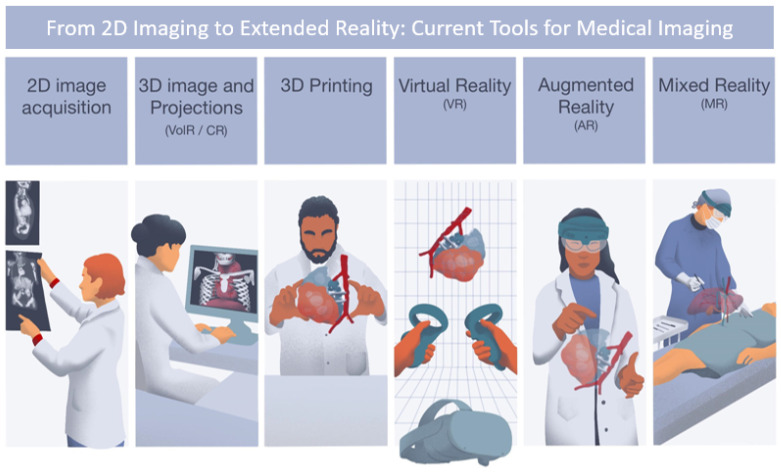
Different visualization technologies for medical imaging, from 2D imaging, 3D projections, and 3D printing to extended reality: VR, AR, and MR.

Nowadays, Computed Tomography (CT), Magnetic Resonance Imaging (MRI), and Positron Emission Tomography (PET) represent the main imaging tools in the diagnostic process of pediatric tumors. Post-processing techniques, including three-dimensional imaging projections such as Volume Rendering (VolR) [12], Cinematic Rendering (CR) [13], 3D modeling techniques, 3D printing (3DP), Virtual Reality (VR), and Augmented Reality (AR), are also becoming popular with publications focusing on their impact on surgical planning [10,14,15,16,17,18,19]. These projection techniques and the new post-processing technologies provide three-dimensional visualization, allowing a new approach in exploring vascular structures and anatomical tumor involvements (See Figure 1) [20]. These technologies allow for mass personalization of diagnosis and treatment, enhanced visualization of anatomical relations, and guidance in the surgical field.

For instance, VR is a technology that creates a simulated environment that can be experienced through a headset or other devices. This three-dimensional environment is computer-generated and can be interactive, providing an immersive and quasi-realistic experience. In healthcare, VR can be used for simulations of medical procedures, surgical planning, patient education, and pain management [21,22,23]. For surgical planning or simulation, VR models are generated with special software that aids the generation of the three-dimensional environments (normally based on patient images), which are based on Digital Imaging and Communications in Medicine (DICOM) data of Computed Tomography (CT) or MRI scans. AR models are generated with specific software, following the same workflow as VR. The main difference between AR and VR is that VR is visualized in a fully immersive headset, while AR uses a headset with mounted cameras or sensors and a transparent screen allowing the user to visualize and interact with the surroundings. AR technology overlays digital information on top of the real world, allowing for interaction between the digital and physical worlds, but not anchoring the digital data to physical parts. In healthcare, AR can be used for medical education, surgical navigation, and patient education and communication [24,25,26]. Finally, Mixed Reality (MR) combines elements of VR and AR to create an environment that blends the physical and digital worlds, allowing superimposing and anchoring digital information on top of physical parts, with the capacity to interact with it visually being only through the MR headset. VR, AR, and MR, altogether known as extended reality (XR), represent a new frontier in medical education and open opportunities for new tools to support diagnosis and treatment [27]. Quero et al. studied the impact of XR technologies in liver oncology surgeries, showing challenges for MR accuracy with soft tissue, reporting registration and digital–physical anatomy anchoring due to organ deformations during patient breathing or movements [28]. Vervoom et al., after a literature review on the use of MR in interventional practice, concluded that the main MR applications so far were focused on preoperative planning, intraoperative and interventional guidance, and clinical education. The reported experience has been positive so far, showing benefits in surgical efficiency. However, important limitations continue to exist regarding the head-mounted display’s comfort, cost, physical and digital smooth interaction, the need for integration with robots and surgical microscopes, and more evidence with larger cases [29]. This is in accordance with other authors [30]. XR technologies, together with 3D modeling and 3D printing, are increasingly present in the management of surgical oncology, with evidence demonstrating positive impacts in clinical outcomes through surgical planning, training, and intraoperative guidance [13,31].

Due to these characteristics, these technologies may become good tools for adoption in the pediatric surgical oncology field. However, existing examples in the published pediatric literature regarding the application of 3D imaging reconstruction, 3D modeling, and 3D printing have focused mainly on bone-based specialties and cardiovascular anomalies [32,33,34,35,36,37]. Urology and kidney oncological treatments using XR or 3D modeling and printing technologies are the most described in the literature, as well as orthopedic and lung cancers. However, most cases focus on adult patients and very few examples exist in pediatrics [30,31,32,33,34,35,36,37,38,39,40].

Therefore, in this work, we present the main innovative imaging reconstruction techniques (VolR, CR), 3D modeling technologies (CAD, VR, AR), and 3D printing applications through the analysis of three real cases of the most common and surgically challenging pediatric tumors: abdominal neuroblastoma, thoracic inlet neuroblastoma, and a bilateral Wilms tumor candidate for nephron-sparing surgery. We explain the complete workflow from image acquisition to final model representation for surgical planning, surgical training, education, or simulation. We also present the technical challenges and limitations of each approach. Finally, we provide an evaluation of the advantages and pitfalls of each approach for the differential diagnosis and treatment.

## 2. Materials and Methods

### 2.1. New Digital Workflow from Image Acquisition to Final Model Representation

CT and MRI represent the main imaging tools in the diagnostic process of pediatric tumors. MRI is the most-used acquisition technique for soft tissue, due to its improvement in tissue visualization and characterization without ionizing radiation, while CT is more used for bone structures. However, in complex pediatric cases, both CT and MRI are frequently combined to improve the imaging reconstruction accuracy.

**Figure 2:**
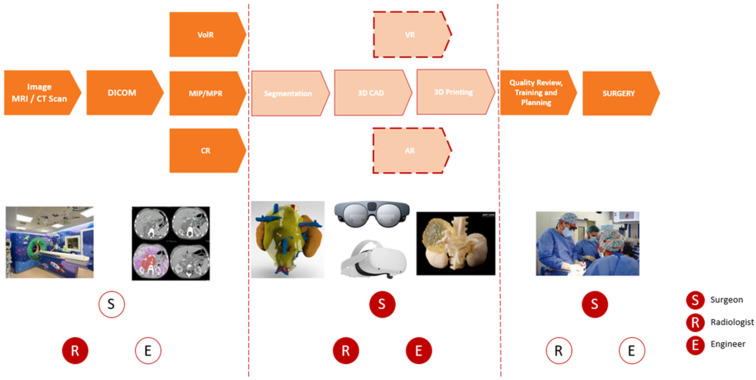
Workflow of the surgical planning and specialists involved in each phase.

In recent years, new post-processing techniques, such as VolR, CR, 3DP, VR, or AR, have become popular and allow the first 3D visualization of the anatomy. These new techniques require the addition of several steps in the digital workflow of surgical planning. These new processes also require the collaboration of multidisciplinary teams composed of radiologists, engineers, and surgeons. The outline of the new digital workflow is defined and presented in the flowchart below, with the new steps in the surgical planning process shown in light orange (see Figure 2).

### 2.2. Case Presentation

To study the advantages and disadvantages of the new imaging post-processing techniques and technologies in performing a proper differential diagnosis when planning complex pediatric oncology cases, technologies were studied by means of three common pediatric cancers: abdominal neuroblastoma, thoracic inlet neuroblastoma, and a Stage V bilateral Wilms tumor for nephron-sparing surgery. Information about each case is summarized in Table 1.

Case #1 is a 3-year-old patient with an abdominal neuroblastoma with Image Defined Risk Factors (IDRF) invading the spinal canal and encasing the abdominal aorta, superior mesenteric artery, and both the right and left renal arteries. The left and right renal veins ran peripherally to the tumor. Furthermore, the inferior vena cava was displaced anteriorly and in contact with the mass.

Case #2 is an 11-year-old patient who presented with a neuroblastoma located in the left thoracic inlet extending into the left lateral neck, centered at levels III–IVb and partially surrounding the left supra-aortic trunks.

Case #3 is a 2-year-old patient with bilateral Wilms tumor (Stage V). On the left side, the 56 mm × 49 mm × 46 mm tumor was located on the medial side of the kidney, while on the right side it measured 56 mm × 49 mm × 46 mm and the tumor reached the renal hilum, causing minimal dilation of the intrarenal excretory system. The right side will be the one shown in this paper.

Imaging 3D renderings, 3D reconstructions, and 3D printed models were performed for research purposes to study the best surgical planning approach and surgical planning purposes.

The following sections describe the different steps of the workflow.

### 2.3. Image Acquisition

In complex pediatric surgical oncology cases, in which soft tissue and bone are involved and are important to help assess the differential diagnosis and treatment approach, both CT and MRI are needed.

For the assessment of the vascular–tumor relationships in the Case #1 patient, an abdominal CT angiography with contrast was performed.

In Case #2, the patient underwent a neck and thoraco-abdominal CT scan with endovascular venous phase contrast (split-bolus technique).

Finally, for Case #3, a combination of MRI and CT images was required. An abdominal MRI was performed, acquiring T1 and T2 sequences in all three planes of space, along with diffusion-weighted images. The abdominal and pelvic angio-CT was performed using the split-bolus technique. The CT scan was performed using a Philips iCT 256 slice scanner.

After image acquisition, post-processing was performed to obtain the imaging projections and 3D models for surgical planning and differential diagnosis assessment. Medical imaging post-processing and planning techniques can be divided into three types: (1) imaging projections (Multiplanar Reconstruction (MPR), Volume Rendering (VolR), Maximal Intensity Projection (MIP), or Cinematic Rendering (CR)); (2) Computer Aided Design (CAD) 3D models, which can be viewed using specialized software and devices such as screen, VR, or AR headsets, and (3) 3D printed models.

### 2.4. Medical Imaging Projections

Medical imaging projections or renderings are visual representations of medical imaging data, such as CT scans, MRI scans, and PET scans. They are used to create detailed and accurate 3D visualizations of internal structures and organs, such as tumors, blood vessels, and bones, among others.

Medical imaging renderings were created using various techniques, such as Volume Rendering, Surface Rendering, or Multiplanar Reconstruction. All of these techniques were performed using Intellispace Portal from Philips© (Amsterdam, the Netherlands).

#### 2.4.1. Multiplanar Reconstruction

MPR is a method for displaying three-dimensional (3D) datasets from two-dimensional images acquired from different planes (axial, sagittal, and coronal). It allows the production of sectional images, such as original two-dimensional coronal, sagittal, and oblique images. Curved-MPR reconstructs sectional images perpendicular to a specific curved line made by the user.

#### 2.4.2. Maximal Intensity Projection

MIP is a technique that projects the brightest voxel (3D pixel) along a particular viewing direction onto a 2D image plane. This creates a 2D image that highlights the most intense structures in the 3D volume, such as bones or blood vessels. MIP images are useful for identifying and visualizing specific structures and for detecting lesions or abnormalities.

#### 2.4.3. Volume Rendering

VolR is a type of data visualization technique which creates a three-dimensional representation of data. It uses the data from all voxels in the 3D volume to create a 3D image that provides a more accurate representation of the underlying structures. VolR images are created by computing the intensity of light passing through the volume at each point, and can be used to create images that are more realistic and show the relationships between different structures. CT and MRI data are frequently visualized with Volume Rendering in addition to other reconstructions and slices. This technique can also be applied to tomosynthesis data

#### 2.4.4. Cinematic Rendering

CR is a 3D rendering algorithm that simulates the propagation and interaction of light rays as they pass through the volumetric data, producing a photorealistic representation of 3D images. CR works by using advanced algorithms and mathematical models to simulate the physical properties of light and matter, creating highly realistic images and videos. The process typically begins by creating a 3D model of the object or scene that is to be rendered. This model is then lit and shaded using various techniques such as global illumination which simulate the way that light bounces and reflects off surfaces, and ambient occlusion, which adds depth and realism to shadows.

Once the lighting and shading have been applied, the image is then rendered, or computed, using powerful computer hardware. This process can take several minutes or even hours, depending on the complexity of the scene and the desired level of detail. Finally, post-processing techniques such as color correction and compositing are used to enhance the overall visual quality of the final image or video.

### 2.5. Image Segmentation

Another post-processing technique is the transformation of the DICOM (Digital Imaging and Communications in Medicine) file obtained from the CT and MRI to a 3D surface anatomical model. This process involves DICOM data segmentation by extracting the desired structures or areas of interest from the surrounding tissue, which is performing using specific software tools. The segmented model will be exported to a new CAD file format to enable its manipulation. It can be converted to three types of formats: OBJ (Object), 3mf (3D Manufacturing Format), or STL (Standard Triangle Language) file. STL is a file format commonly used in CAD modeling and 3D printing that represents the surface of a 3D object as a series of triangles. This step allows for the further manipulation, design, and preparation of the final 3D model and is necessary before the 3D virtual simulation, 3D printing, or preparation of VR or AR models.

The image segmentation in the three presented cases was carried out by an expert 3D planning biomedical engineer under the supervision of an expert radiologist to extract the anatomy to be used in the 3D planning and printing. A semi-automatic segmentation was performed using the IntelliSpace Portal© v12 software from Philips©. The 3D surface STL model was obtained by the addition of the DICOM segmented masks’ areas of interest and exported to an STL file.

### 2.6. CAD Design and 3D Modeling

The 3D STL files obtained were transferred to the 3D planning unit, where 3D planning biomedical engineering experts prepared the 3D virtual simulation CAD models with the anatomy of interest for each case. This process was performed using Materialise Mimics version 25.0 Medical© software (Belgium). The process of 3D planning in the three cases was iterative, performed with the validation and supervision of senior pediatric oncology surgeons and senior radiologists. After validation of the anatomical 3D model, the simulation of the surgical approaches started with the calculation of the potential tumor resection volumes for each case.

### 2.7. Three-Dimensional Virtual Simulation and VR

To enhance communication between the 3D planning biomedical engineers and the pediatric oncology surgeons, 3D visualization of the cases was provided to permit mobile device access and remote visualization. This was achieved using the Materialise Mimics Viewer© Version 2.2.43.5. Surgeons could assess the advances in the planning process from a mobile phone or tablet while interacting with the 3D rendering, performing calculations, or taking notes on top of the 3D models. This process allowed surgeons to provide advice to biomedical engineers or other surgeons involved in the case.

### 2.8. VR and AR

The final 3D models, clinically validated by the senior surgeons of each case, were prepared by the 3D planning biomedical engineers to be seen through VR or AR headsets. This was achieved using Materialise Mimics Viewer© VR and AR extensions. The VR headset used in all cases was Oculus Meta Quest 2 headset (Meta Platforms Ireland Limited, Dublin, Ireland).

### 2.9. Three-dimensional Printing

After the 3D virtual simulation was performed, the anatomy to be included in the 3D printed anatomical models was decided. Three-dimensional printing of the anatomical models started by defining the anatomical regions of interest and the hardness, color, and accuracy needed in each case. A senior oncology surgeon defined these requirements based on the clinical diagnostic and treatment assessment needs. All cases were manufactured using J5 MediJet© material jetting technology (Stratasys, Eden Prairie, MN, USA). The printing parameters and materials used for each case are summarized in Table 2.

All cases were manufactured in-house at the 3D4H Unit of SJD Barcelona Children’s Hospital.

## 3. Results

In this work, we want to highlight the main advanced imaging reconstruction and post-processing techniques for evaluating the differential diagnosis, surgical planning, and treatment assessment through the analysis of three complex pediatric oncology cases using DICOM reconstruction techniques (MPR, VolR, CR), 3D virtual simulation, VR and AR visualizations, and 3D printing.

### 3.1. Three-Dimensional Planning Models: From Imaging Projections to VR and 3D Printed Models

**Figure 3:**
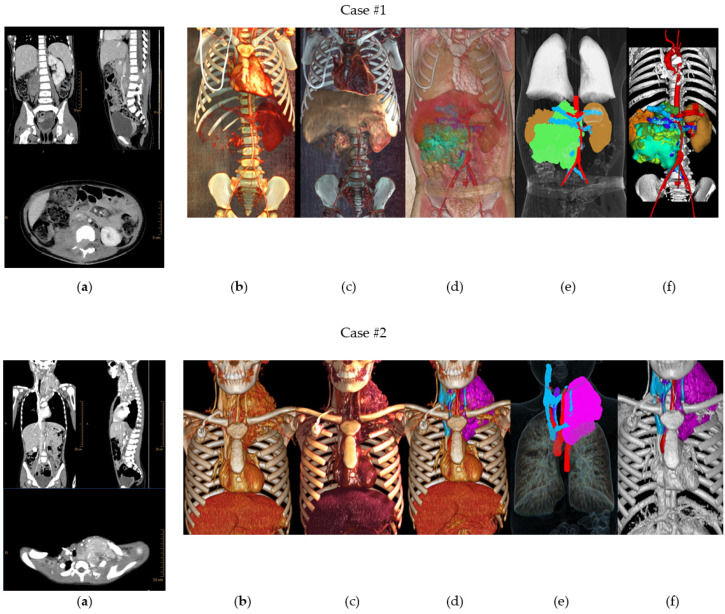

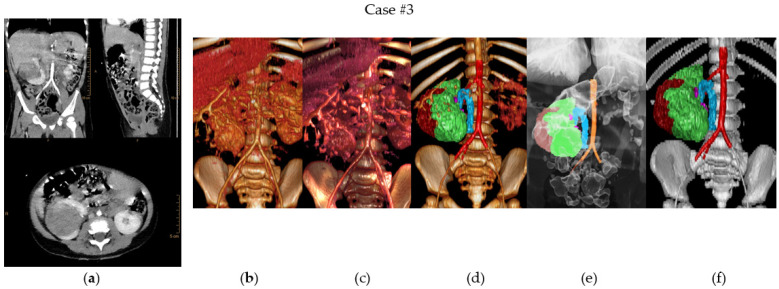
Different imaging post-processing techniques: (**a**) MPR, (**b**) VolR, (**c**) CR, (**d**) combination of VolR with CAD, (**e**) combination of airway with segmentations, and (**f**) CAD models.

Figure 3 shows the different imaging post-processing techniques (MPR, VolR, CR, combination of VolR with CAD, combination of airway with segmentations, and CAD models) developed in each case.

#### 3.1.1. Two-Dimensional Projection Renderings

**Figure 4:**
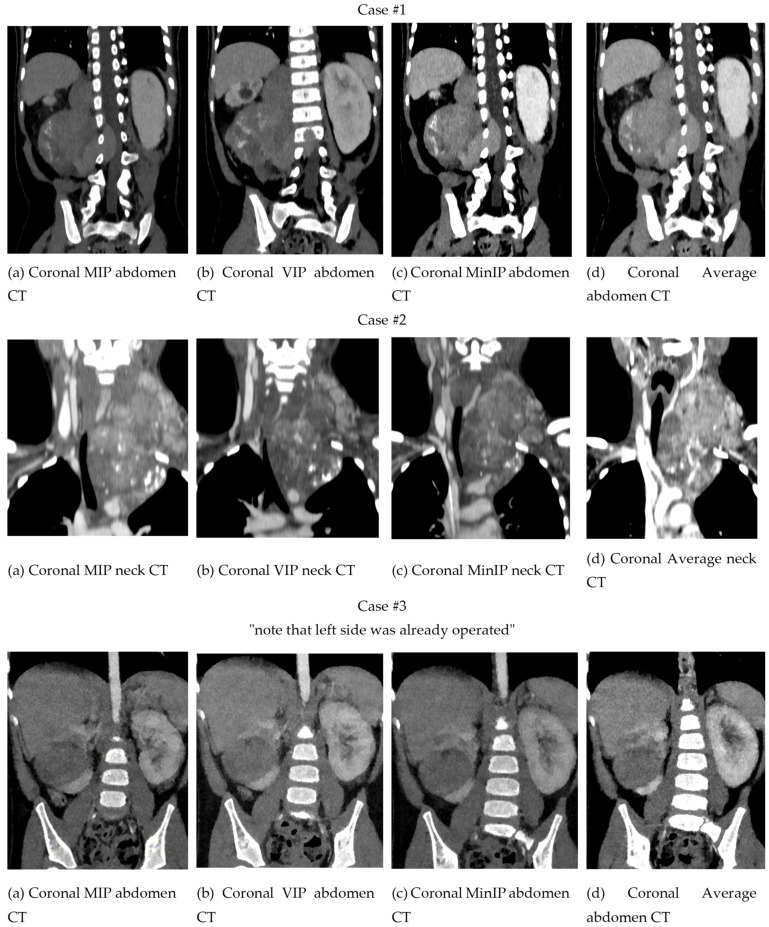
Comparison between different projections with (**a**) Maximal Intensity, (**b**) Volumetric Intensity, (**c**) Minimal Intensity, and (**d**) Mean reconstructions.

The comparison between different projections with Maximal Intensity, Volumetric Intensity, Minimal Intensity, and Mean reconstructions is presented in Figure 4. In this example, of a patient with a neuroblastoma with invasion of the spinal canal, it can be observed how each projection modifies the visualization of the structures. In this sense, the thickness of the slice is the key, since it can superimpose elements of greater or lesser density and modify the interpretation of the extension to the spinal canal.

#### 3.1.2. Volume Rendering and Cinematic Rendering

**Figure 5:**
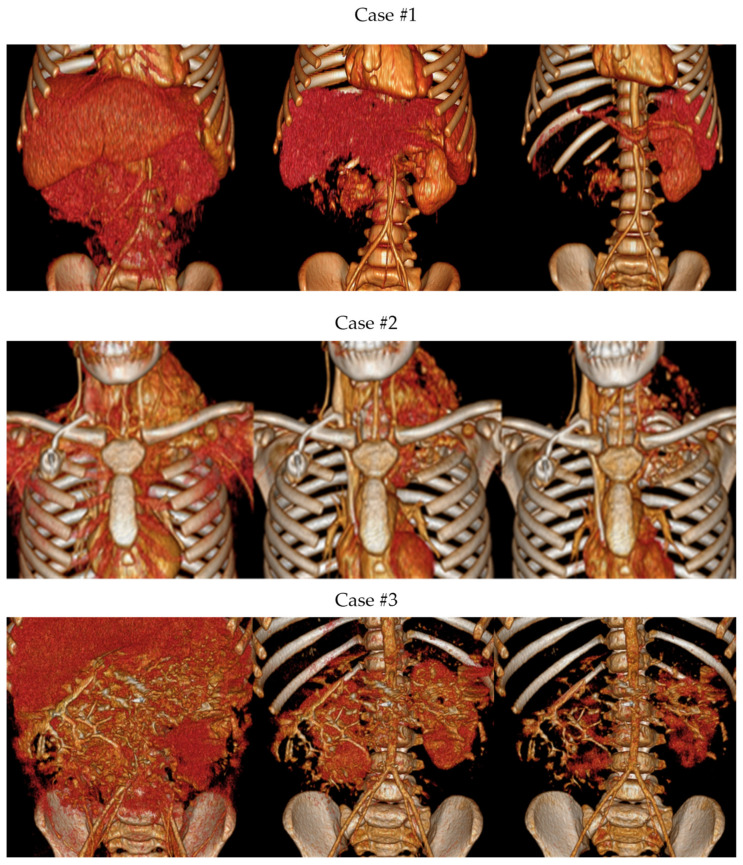
Volume Rendering of the three cases at different window levels.

As shown in Figure 5, VolR represents a good technique for visualizing large volumes of data generated by modern CT/MRI scanners in three-dimensional space. The different aspects of the dataset can be interactively explored in the 3D VolR window. This technique allows for clear 3D image viewing for surgical planning and the exploration of anatomical relations, as it provides a much more detailed view of the patient’s anatomy than 2D images alone. However, it does not permit proper interaction with the image, being a projected visualization instead of an object. Moreover, it does not represent the true anatomical relations, thus affecting diagnostic viability; therefore, it is not a valid solution for radiological evaluation if used as the only diagnostic technique.

**Figure 6:**
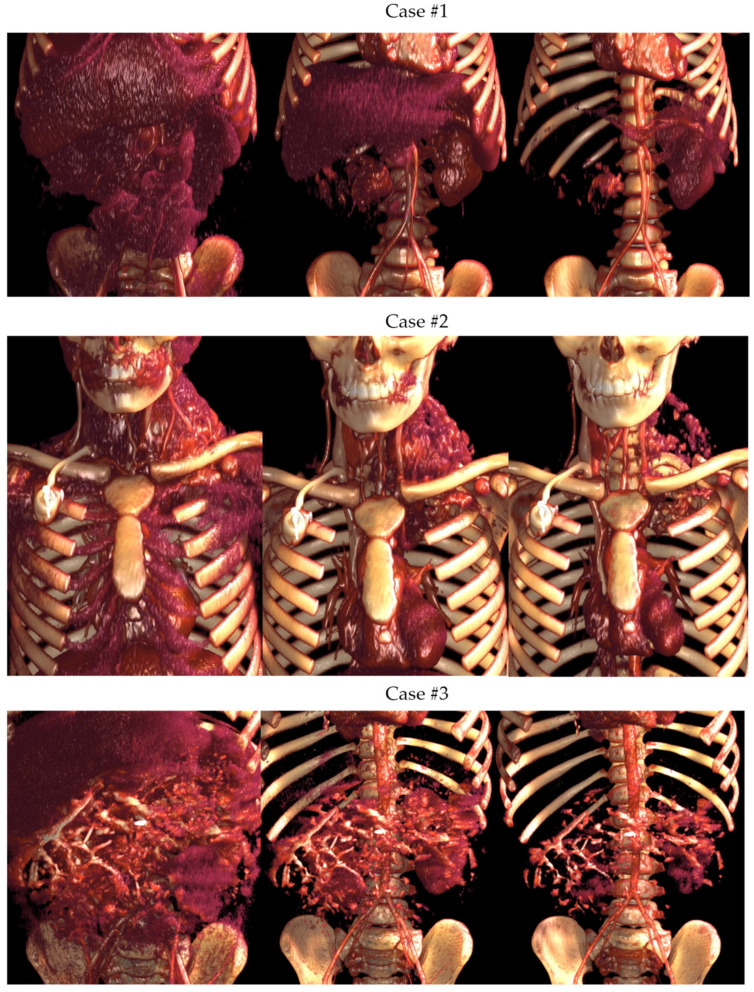
Cinematic Rendering of the three cases at different window levels.

On the other hand, CR provides a lifelike visualization of the anatomy, going beyond the representation of VolR for surgical planning and anatomy exploration with highly realistic views for imaging diagnosis (see Figure 6). This can provide a realistic and immersive view of the patient’s anatomy, allowing surgeons to better understand the relationships between different structures. However, as happens with VolR, it does not allow proper interaction with the image, being a projected visualization instead of an object, affecting the interpretation of specific measurements in anatomical relations. For this reason, these two techniques are recommended to be used as a complement to 2D image projections when used for radiological evaluation.

#### 3.1.3. Three-Dimensional Virtual Simulations (CAD Models)

**Figure 7:**
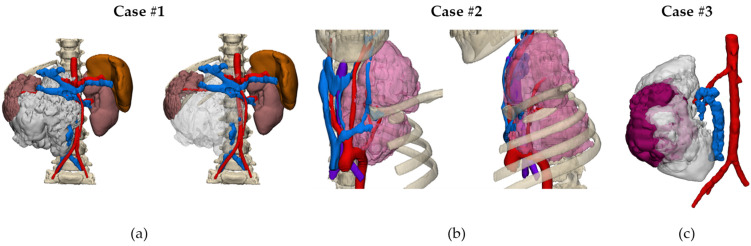
Representations of the CAD files of the 3 cases: (**a**) abdominal neuroblastoma, (**b**) cervical neuroblastoma, and (**c**) Wilms tumor.

Figure 7 shows the 3D virtual CAD models of the three cases. These results were shared with the clinical team by using a virtual representation accessible from different devices using Mimics Viewer Version 2.2.43.5 from Materialise© (Belgium, Leuven).

**Figure 8:**
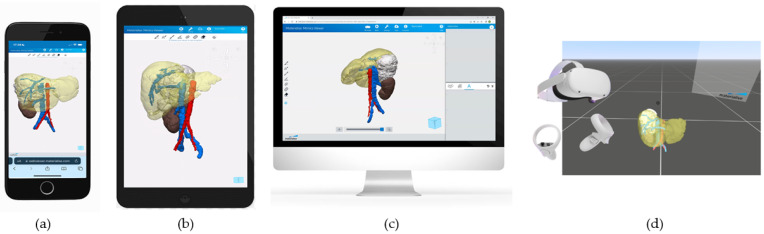
Four different digital visualization options: (**a**) smartphone, (**b**) tablet, (**c**) computer, and (**d**) VR.

The virtual simulations can be visualized with three different options: smartphone (Figure 8a), tablet (Figure 8b), or computer (Figure 8c).

#### 3.1.4. Virtual and Augmented Reality

The 3D CAD files used in the 3D virtual simulations can also be visualized through Virtual Reality and Augmented Reality goggles (Figure 8d). Interaction with the 3D CAD image is performed via the use of two haptic joysticks, allowing for a variety of actions to visualize the models, serving as a tool for understanding the anatomy, for surgical planning, or surgeon training. The main difference between VR and AR is that AR allows for the visualization of the 3D model on top of the real environment, becoming a promising tool to be used during interventions. Being able to visualize the surgical planning, not only through a desktop computer but also through mobile devices such as a smartphone or tablet, or through a VR or AR headset, allows for improved engineer–surgeon communication during the planning process. It also allows the surgeon to review the case at any time, representing one more alternative to those already available for case planning or review.

#### 3.1.5. Three-Dimensional Printing

**Figure 9:**
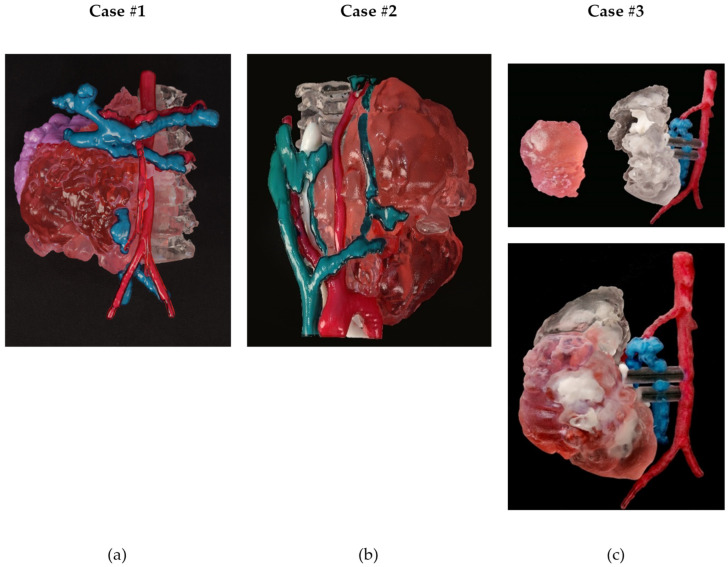
The 3D printed models of the three cases. (**a**) Case #1: Abdominal neuroblastoma (**b**) Case #2: Thoracic Inlet neuroblastoma (**c**) Case #3: Right side of a bilateral Wilms’ tumor (nephron sparing surgery).

Figure 9 shows the 3D printed multicolor models of the three cases.

The anatomical model of Case #1 (Figure 9a) consisted of a single model combining all the anatomical parts. Bone was printed using a transparent material, which allowed an accurate analysis of the part of the tumor that had invaded the spinal canal. The veins were blue, the arteries were red, the kidney was purple, and the tumor was printed in light red.

In the anatomical model of Case #2 (Figure 9b), bone was also transparent, the veins were blue, the arteries were red, the kidney was purple, and the tumor was printed in light red.

In the 3D printed model of Wilms tumor (Case #3, right side only) (Figure 9c), the kidney was printed using a transparent material, while the calyces were white, the veins were blue, the arteries were red, and the tumor was printed in light red. Additionally, the kidney and the tumor models were printed separately to better visualize the volume of the tumor and the healthy kidney portion, and observe the calyces that were in contact with the kidney.

### 3.2. General Considerations

**Figure 10:**
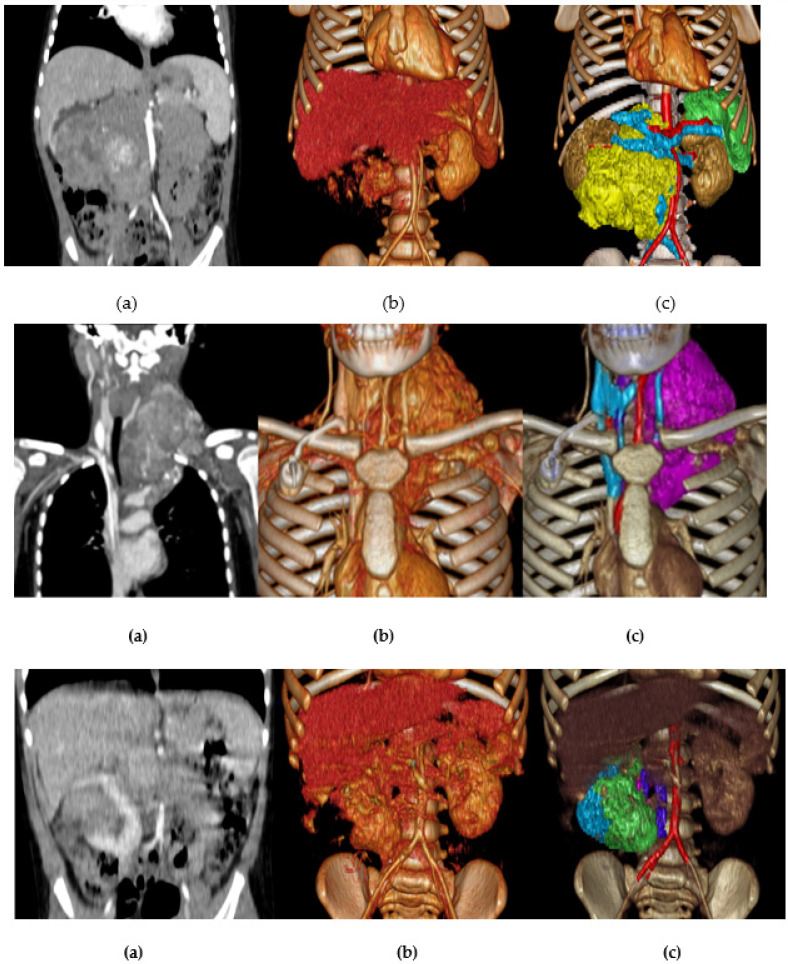
Comparison between different projections modalities of the three cases: (**a**) Coronal MPR, (**b**) Coronal Volume Rendering, and (**c**) Coronal VRT and segmented tissue.

Figure 10 shows a comparison between the different projection modalities of the three cases. The image on the right, combining VolR and segmentation, helps us to identify the tumor faster and more intelligibly, compared to VolR. However, we lose the internal density information.

## 4. Discussion

### 4.1. Advantages and Pitfalls of Each Approach for the Differential Diagnosis and Treatment

Nowadays, CT and MRI 2D imaging projections represent the main imaging tools for radiological evaluation of pediatric oncological cases. In complex cases, with challenges in the identification of anatomical relations, multimodality imaging is used to allow reliable diagnosis correlated, when needed, with guided biopsies [41]. Modern post-processing techniques together with contrast agents allow for a detailed evaluation of vasculatures, delineate lesions, and better characterization of the anatomic relations [42]. This is a very important aspect for surgical planning, as the surgeon needs to be prepared as much as possible to foresee and be prepared for challenging situations and potential complications. Recent advances in intraoperative and preoperative imaging and progress in visualization tools bring new insights into pediatric tumors to guide therapeutic decisions and prognosis in different pediatric surgical oncology areas and locations, including thoracic, brain, urology, and abdominal surgery [43,44,45,46,47].

Table 3 summarizes the cost, time needed, main advantages, and recommended indication for each image post-processing technique.

MPR represents true anatomy relations and is indicated for radiological evaluation, although it may be less visually friendly for non-trained professionals due to its 2D projections.

CR and VolR need accurate definition of the window level to properly visualize the full anatomy. This may be an issue when exploring soft tissue such as tumor and vessels, making it challenging in some cases to properly visualize both at the same time (see Figure 5).

To obtain 3D printed models, VR, and AR, a segmentation of the DICOM image of the patient is needed, which represents more resources and time. Added to that, 3D printed models have a long printing time, which can exceed one day in complex anatomy depending on the anatomical dimensions. Thus, 3D physical models could be justified in cases where tactile feedback is needed. VR and AR allow surgeons to visualize the different relationships of the anatomy to obtain a better understanding of complex cases.

In the case of 3D printing, the cost is high but it depends on the anatomical parts, and their dimensions, that are included in the model. For an NB, the average cost is EUR 461, while for a Wilms tumor case the cost is approximately EUR 130.

### 4.2. Clinical Relevance

#### 4.2.1. From a Surgical Point of View

Access to 3D technologies provides the opportunity to better understand the anatomical aspects of complex tumors. Gold standard imaging techniques for studying solid tumors are 2D planar; thus, the surgeon should “mentally create” a 3D model for planning the procedures. The 3D technology allows that step to be skipped, opening a wide range of possibilities, from easily viewing, measuring, and modifying a 3D model with the use of Virtual Reality goggles, to printing a model and using it in the operating room while the intervention is being performed. Every effort should be made for facilitating surgical intervention in complex cases, and the inclusion of expert engineers has been a valuable addition to surgical teams around the world, as their computing and design skills improve conventional imaging, taking surgical planning to the next level. However, among the disadvantages, the process of obtaining 3D models is relatively slow, especially if 3D printing is needed. Future directions of 3D technologies are promising, due to the progressive use of their different applications and the combination with new technologies such as artificial intelligence. Multiple studies around the world are being conducted, with the objective of generating high-quality evidence that will further define the indications and potential of 3D viewing and printing for surgical planning, simulation, education, and other purposes. From the surgical point of view, the use of new technologies such as 3D imaging, 3D printing, and Virtual Reality is an asset for the planning of complex pediatric oncological surgeries. With the evidence generated and the barriers to adoption overcome, these new technologies have the potential to alter the traditional workflow in the planning and surgical practice of complex oncological surgery, allowing them to be implemented in routine surgical practice. However, as presented in the present work, many existing technologies bring different advantages and disadvantages that can be of value depending on the clinical need. For instance, 3D printing is an expensive technology but brings great value when tactile and hands-on training is needed. VR constitutes a cheaper alternative when a 3D visualization and anatomy interaction can be of help. Finally, 3D projections such as Volume Rendering or Cinematic Rendering could help complement the classic imaging techniques. To unlock the full potential of these technologies, more research is needed, with prospective evaluations of large patients’ cohorts conducted by pediatric surgeons for different pediatric cancer types.

The authors of the present work will continue to work with the new digital workflow for the planning of complex cases and with the aim of continuing to generate the necessary evidence.

#### 4.2.2. From a Radiological Point of View

At the radiological level, the findings show the added value of the different technologies. Volumetric renderings allow for quick mapping of the findings. However, as tumor tissue may have a similar density to other soft tissue, they may occasionally be difficult to differentiate when using basic rendering density-based reconstructions. Therefore, the segmentation of target tissue can be helpful if relationships need to be quickly and unambiguously shown to the surgeon or patient. One of the advantages of virtual models is the possibility of selecting the specific tissue to be shown, as well as providing them with properties such as transparency, which makes them very useful for understanding complex relationships, as well as the depth of lesions. However, despite the limitations, the reconstructions and 3D models allow the radiologist to have general information about the anatomy and general volume of the lesion, as well as its spatial relationships, even allowing the approach route to be simulated. In short, the different models offer us greater insight into the anatomical relationships of the tumor, which can be complex. Aspects related to tumor type, its location, the time required for reconstruction, and the anatomical structures to show are the key when choosing one technique or another.

### 4.3. Limitations of the Technique

There are several limitations to medical imaging rendering techniques, including:

- Image Quality: The quality of the final rendering can be affected by the quality and resolution of the original imaging data, as well as the specific algorithms and techniques used in the rendering process.
- Patient Variability: Different patients can have different anatomies, tissue densities, and other factors that affect the accuracy and reliability of the renderings.
- Interobserver variability: Different radiologists or physicians may interpret the images differently, leading to variations in the diagnosis or treatment plan.
- Time-consuming: The rendering process can be time-consuming and may require specialized software and hardware, which can be costly.
- Limited applicability: The renderings may not be suitable for all types of medical imaging, such as nuclear medicine or ultrasound, which have different modalities.
- Training: Medical professionals need to be trained to use the software and interpret the images correctly.
- Costs: The procurement of 3D printing technologies such as 3D printer, VR or AR headsets, or software packages to implement advanced projection techniques (VolR or CR), as well as the resources needed for bioengineers and setting an in-house lab, may be a limitation for the adoption of the presented techniques.
- Regulation: Complying with local regulations to use these new technologies for surgical planning and treatment can be a barrier to their implementation, mainly due to the time and resources needed.
- Lack of Validation: Some of the techniques used in medical imaging rendering have still to be validated for clinical use.
- Privacy and security: Storing and sharing of medical imaging data can raise privacy and security concerns.

Despite these limitations, medical imaging renderings can be a valuable tool for diagnosis and treatment planning and can help to improve patient outcomes. With the development of technology, the limitations will be minimized in the future.

CR, 3DP, VR, and AR represent an extra cost and time to the existing workflow. Moreover, more evidence is needed in the use of CR, 3DP, VR, and AR as an improvement to existing 3D imaging visualizations for the differential diagnosis and treatment assessment of pediatric oncology cases.

### 4.4. Limitations of This Work

This work presents the potential of 3D imaging and modeling in pediatric surgical oncology based on three different cases as examples, presenting the advantages and disadvantages of each technology. It also presents a new digital workflow for surgical planning and treatment aid and the main actual challenges for implementation. However, for the full adoption of these new technologies, there is a need for clinical research with larger cohorts to quantitively demonstrate the impact of each technology and validate the recommended applications presented.

## 5. Conclusions

Although CT and MRI 2D imaging techniques represent the main imaging tools in the diagnostic process, the advances in recent years of new techniques such as MPR, CR, VolR, VR, AR, and 3D printing technologies make them a good alternative for complex oncological cases in which anatomical vascular and tumor relations need to be well defined. Knowing the advantages and limitations of each technique will help establish an optimal approach according to the clinical need. For instance, 3D printing brings the advantage of having a physical model, providing the haptic touch, and a perception of the real dimensions and anatomical relations. Furthermore, 3D printed models allow practicing surgical techniques on the physical model, being an important aspect for the education of students. Three-dimensional printed models could also facilitate patient–physician communication. VR and AR help in seeing the 3D image of the patient’s anatomy by providing a 3D immersive view, with the possibility of interacting closely with it. These three technologies are becoming increasingly popular, not only in surgical planning but also for training and simulation. Yet, the cost of each of these technologies must be taken into account when deciding which one to use. Due to the actual high cost of realistic 3D printed models, their use is only prioritized in the most complex and necessary cases. In the near future, new technologies will probably become integrated into normal clinical practice, taking advantage of their main characteristics in meeting the needs of each specific application. However, for the full adoption of these new technologies, structured validation is needed. This implies that further research and development of clinical trials and testing are needed.

## Figures and Tables

**Table 1:** Information about the presented cases.

	Age (Years)	Sex	Diagnosis	Surgical Approach
Case #1	3	F	Abdominal neuroblastoma	Laparotomy
Case #2	11	F	Thoracic Inlet neuroblastoma	Trap Door
Case #3	2	F	Bilateral Wilms tumor (nephron-sparing surgery)	Bilateral Transverse Laparotomy

**Table 2:** The 3D printing parameters and materials of the 3D printed models of the three cases presented.

Case	Technology	Printer	Printing Time	Cost	Model Materials	Support Material
1	Material Jetting	J5 MediJet©	1 day 12 h 36 min	EUR 486,70	VeroCyan™V for veins, VeroMagenta™V for arteries, Med610 for bone; tumor was printed with a combination of VeroMagenta™V + Elastico Clear with 60A shore hardness	SUP710™ (Water removable)
2	Material Jetting	J5 MediJet©	24 h 21 min	EUR 435,51	VeroCyan™V for veins, VeroMagenta™V for arteries, Med610 for bone, DraftWhite for airway; tumor was printed with a combination of VeroMagenta™V + Elastico Clear with 60A shore hardness	SUP710™ (Water removable)
3	Material Jetting	J5 MediJet©	10 h 56 min	EUR 130,33	VeroCyan™V for veins, VeroMagenta™V for arteries, Med610 for the kidney, DraftWhite for calyces; tumor was printed with a combination of VeroMagenta™V + Elastico Clear with 60A shore hardness	SUP710™ (Water removable)

**Table 3:** Showing a comparison of each studied technology, their cost of implementation, average time needed per case, main advantages, and recommended applications.

Image Post-Processing	Cost	Time	Virtual Visualization	Tactile Planning	Main Advantages	Recommended Indication
MIP/MPR	Low	Low (10 min)	Yes	No	True anatomy relations. Vascular invasion.	Radiological evaluation.
VolR	Low	Low (10 min)	Yes	No	General 3D relationships. Good tissue differentiation helps understanding.	3D fast overview. General understanding. Bone fractures. Bone–vascular anatomy.
CR	Low	Low (10 min)	Yes	No	Realistic 3D visualizations. Good tissue differentiation helps understanding of anatomical parts. Enhanced the overall visual quality.	3D high-realism overview. General understanding. Bone fractures. Bone–vascular anatomy.
3D CAD	Mid	Mid (1 h)	Yes	No	Optional to visualize. Volume measurements (% tumor removal).	Surgical planning and simulation. Post-surgical treatment information.
3D PRINT	High	High (from 11 h to 1 day and 12 h)	Yes	Yes	Tactile feeling and practice. Patient experience and comprehension. Patient-specific surgical tool generation.	Surgical planning. Patient-specific surgical tool fabrication. Education and training. Patient communication.
VR	Mid	Mid (1 h 15 min)	Yes	Yes	Immersive 3D experience in an interactive virtual space.	Surgical planning. 3D overview—general understanding Education and training. Patient communication.
AR	Mid	Mid (1 h 15 min)	Yes	Yes	Interactive mixed reality with virtual and physical reality information.	Real-time guidance to surgeons during procedures. Superimposing images to improve understanding.

## Data Availability

Due to the sensitive nature of the data used in this study, the datasets used and/or analyzed during the current study are available from the corresponding author on reasonable request. Imaging data will remain confidential and will not be shared.

## References

[1] Wijnen M. (2022). Innovations in pediatric surgical oncology. J. Pediatr. Surg..

[2] Goldstein S.D., Heaton T.E., Bondoc A., Dasgupta R., Abdelhafeez A., Davidoff A.M., Lautz T.B. (2021). Evolving applications of fluorescence guided surgery in pediatric surgical oncology: A practical guide for surgeons. J. Pediatr. Surg..

[3] Jacobson J.C., Scrushy M.G., Gillory L.A., Pandya S.R. (2023). Utilization of robotics in pediatric surgical oncology. Semin. Pediatr. Surg..

[4] Lazar J.F., Hwalek A.E. (2023). A review of Robotic Thoracic Surgery Adoption and Future Innovations. Thorac. Surg. Clin..

[5] Warmann S., Fuchs J., Jesch N.K., Schrappe M., Ure B.M. (2003). A prospective study of minimally invasive techniques in pediatric surgical oncology: Preliminary report. Med. Pediatr. Oncol..

[6] Tejo-Otero A., Buj-Corral I., Fenollosa-Artés F. (2019). 3D Printing in Medicine for Preoperative Surgical Planning: A Review. Ann. Biomed. Eng..

[7] Fox M., Peregrin T. (2016). 3-D printing: Revolutionizing preoperative planning, resident training, and the future of surgical care. Bull. Am. Coll. Surg..

[8] Krauel L., Fenollosa F., Riaza L., Pérez M., Tarrado X., Morales A., Gomà J., Mora J. (2015). Use of 3D Prototypes for Complex Surgical Oncologic Cases. World J. Surg..

[9] Tack P., Victor J., Gemmel P., Annemans L. (2016). 3D-printing techniques in a medical setting: A systematic literature review. Biomed. Eng. Online.

[10] Krauel L., Valls-Esteve A., Tejo-Otero A., Fenollosa-Artés F. (2021). 3D-Printing in surgery: Beyond bone structures. A review. Ann. 3D Print. Med..

[11] Privitera L., Paraboschi I., Dixit D., Arthurs O.J., Giuliani S. (2021). Image-guided surgery and novel intraoperative devices for enhanced visualisation in general and paediatric surgery: A Review. Innov. Surg. Sci..

[12] Aimar A., Palermo A., Innocenti B. (2019). The Role of 3D Printing in Medical Applications: A State of the Art. J. Healthc. Eng..

[13] Wake N., Nussbaum J.E., Elias M.I., Nikas C.V., Bjurlin M.A. (2020). 3D printing, augmented reality, and virtual reality for the assessment and management of kidney and prostate cancer: A systematic review. Urology.

[14] Calhoun P.S., Kuszyk B.S., Heath D.G., Carley J.C., Fishman E.K. (1999). Three-dimensional volume rendering of spiral CT data: Theory and method. Radiographics.

[15] Eid M., De Cecco C.N., Nance J.W., Caruso D., Albrecht M.H., Spandorfer A.J., De Santis D., Varga-Szemes A., Schoepf U.J. (2017). Cinematic rendering in CT: A novel, lifelike 3D visualization technique. Am. J. Roentgenol..

[16] Dappa E., Higashigaito K., Fornaro J., Leschka S., Wildermuth S., Alkadhi H. (2016). Cinematic rendering—An alternative to volume rendering for 3D computed tomography imaging. Insights Imaging.

[17] Ramirez M.E., Pena I.R., Castillo R.E.B., Sufianov A., Goncharov E., Sanchez J.A.S., Colome-Hidalgo M., Nurmukhametov R., Céspedes J.R.C., Montemurro N. (2023). Development of a 3D Printed Brain Model with Vasculature for Neurosurgical Procedure Visualisation and Training. Biomedicines.

[18] Prunoiu V.-M., Popa D., Serbulea M.-S., Bratucu E., Simion L., Bratucu M.N. (2022). Augmented Reality in Surgical Oncology. A Literature Review. Chirurgia.

[19] Fitski M., Meulstee J.W., Littooij A.S., van de Ven C.P., van der Steeg A.F.W., Wijnen M.H. (2020). MRI-based 3-dimensional visualization workflow for the preoperative planning of nephron-sparing surgery in wilms’ tumor surgery: A pilot study. J. Healthc. Eng..

[20] Elshafei M., Binder J., Baecker J., Brunner M., Uder M., Weber G.F., Grützmann R., Krautz C. (2019). Comparison of Cinematic Rendering and Computed Tomography for Speed and Comprehension of Surgical Anatomy. JAMA Surg..

[21] Pereira H.R., Barzegar M., Hamadelseed O., Esteve A.V., Munuera J. (2022). 3D surgical planning of pediatric tumors: A review. Int. J. Comput. Assist. Radiol. Surg..

[22] Capellini K., Tripicchio P., Vignali E., Gasparotti E., Ali L.A., Cantinotti M., Federici D., Santoro G., Alfonzetti F., Evangelista C. 3D Printing and 3D Virtual Models for Surgical and Percutaneous Planning of Congenital Heart Diseases. Proceedings of the 15th International Joint Conference on Computer Vision, Imaging and Computer Graphics Theory and Applications (VISIGRAPP 2020).

[23] Eijlers R., Utens E.M.W.J., Staals L.M., de Nijs P.F.A., Berghmans J.M., Wijnen R.M.H., Hillegers M.H.J., Dierckx B., Legerstee J.S. (2019). Meta-analysis: Systematic review and meta-analysis of virtual reality in pediatrics: Effects on pain and anxiety. Anesth. Analg..

[24] Yeung A.W.K., Tosevska A., Klager E., Eibensteiner F., Laxar D., Stoyanov J., Glisic M., Zeiner S., Kulnik S.T., Crutzen R. (2021). Virtual and Augmented Reality Applications in Medicine: Analysis of the Scientific Literature. J. Med. Internet Res..

[25] Lewis T., Aggarwal R., Rajaretnam N., Grantcharov T., Darzi A. (2011). Training in surgical oncology—The role of VR simulation. Surg. Oncol..

[26] Souzaki R., Ieiri S., Uemura M., Ohuchida K., Tomikawa M., Kinoshita Y., Koga Y., Suminoe A., Kohashi K., Oda Y. (2013). An augmented reality navigation system for pediatric oncologic surgery based on preoperative CT and MRI images. J. Pediatr. Surg..

[27] Barteit S., Lanfermann L., Bärnighausen T., Neuhann F., Beiersmann C. (2021). Augmented, mixed, and virtual reality-based head-mounted devices for medical education: Systematic review. JMIR Serious Games.

[28] Quero G., Lapergola A., Soler L., Shahbaz M., Hostettler A., Collins T., Marescaux J., Mutter D., Diana M., Pessaux P. (2019). Virtual and Augmented Reality in Oncologic Liver Surgery. Surg. Oncol. Clin. N. Am..

[29] Vervoorn M.T., Wulfse M., Van Doormaal T.P.C., Ruurda J.P., Van der Kaaij N.P., De Heer L.M. (2023). Mixed Reality in Modern Surgical and Interventional Practice: Narrative Review of the Literature. JMIR Serious Games.

[30] Wellens L.M., Meulstee J., van de Ven C.P., van Scheltinga C.E.J.T., Littooij A.S., van den Heuvel-Eibrink M.M., Fiocco M., Rios A.C., Maal T., Wijnen M.H.W.A. (2019). Comparison of 3-Dimensional and Augmented Reality Kidney Models with Conventional Imaging Data in the Preoperative Assessment of Children with Wilms Tumors. JAMA Netw. Open.

[31] Arjomandi Rad A., Vardanyan R., Thavarajasingam S.G., Zubarevich A., Van den Eynde J., Pompeu B O Sá M., Zhigalov K., Nia P.S., Ruhparwar A., Weymann A. (2022). Extended, virtual and augmented reality in thoracic surgery: A systematic review. Interact. CardioVascular Thorac. Surg..

[32] Meglioli M., Naveau A., Macaluso G.M., Catros S. (2020). 3D printed bone models in oral and cranio-maxillofacial surgery: A systematic review. 3D Print. Med..

[33] Gehrsitz P., Rompel O., Schöber M., Cesnjevar R., Purbojo A., Uder M., Dittrich S., Alkassar M. (2021). Cinematic Rendering in Mixed-Reality Holograms: A New 3D Preoperative Planning Tool in Pediatric Heart Surgery. Front. Cardiovasc. Med..

[34] Brun H., Bugge R.A.B., Suther K.R., Birkeland S., Kumar R., Pelanis E., Elle O.J. (2018). Mixed reality holograms for heart surgery planning: First user experience in congenital heart disease. Eur. Heart J. Cardiovasc. Imaging.

[35] Lau I., Gupta A., Ihdayhid A., Sun Z. (2022). Clinical Applications of Mixed Reality and 3D Printing in Congenital Heart Disease. Biomolecules.

[36] Ruggiero F., Cercenelli L., Emiliani N., Badiali G., Bevini M., Zucchelli M., Marcelli E., Tarsitano A. (2023). Preclinical Application of Augmented Reality in Pediatric Craniofacial Surgery: An Accuracy Study. J. Clin. Med..

[37] Barcali E., Iadanza E., Manetti L., Francia P., Nardi C., Bocchi L. (2022). Augmented Reality in Surgery: A Scoping Review. Appl. Sci..

[38] Byrd C.T., Lui N.S., Guo H.H. (2022). Applications of Three-Dimensional Printing in Surgical Oncology. Surg. Oncol. Clin. N. Am..

[39] Wijnen M.W., Davidoff A.M. (2021). Minimally Invasive Techniques in Pediatric Surgical Oncology. Surg. Oncol. Clin. N. Am..

[40] Souzaki R., Taguchi T. (2022). Navigational Techniques in Pediatric Surgical Oncology. Pediatric Surgical Oncology.

[41] Schima W., Böhm G., Rösch C.S., Klaus A., Függer R., Kopf H. (2020). Mass-forming pancreatitis versus pancreatic ductal adenocarcinoma: CT and MR imaging for differentiation. Cancer Imaging.

[42] Pandey P., Lewis H., Pandey A., Schmidt C., Dillhoff M., Kamel I.R., Pawlik T.M. (2017). Updates in hepatic oncology imaging. Surg. Oncol..

[43] Montemurro N., Condino S., Carbone M., Cattari N., D’amato R., Cutolo F., Ferrari V. (2022). Brain Tumor and Augmented Reality: New Technologies for the Future. Int. J. Environ. Res. Public Health.

[44] Langdon C., Hinojosa-Bernal J., Munuera J., Gomez-Chiari M., Haag O., Veneri A., Valldeperes A., Valls A., Adell N., Santamaria V. (2023). 3D printing as surgical planning and training in pediatric endoscopic skull base surgery—Systematic review and practical example. Int. J. Pediatr. Otorhinolaryngol..

[45] Liu P., Li C., Xiao C., Zhang Z., Ma J., Gao J., Shao P., Valerio I., Pawlik T.M., Ding C. (2020). A Wearable Augmented Reality Navigation System for Surgical Telementoring Based on Microsoft HoloLens. Ann. Biomed. Eng..

[46] Romao R.L.P., van der Steeg A.F.W., Malek M., Irtan S., Gow K., Ghandour K., Biasoni D., Davidoff A., Pachl M. (2023). Technical advances in the surgical management of Wilms tumors in children. Pediatr. Blood Cancer.

[47] Paraboschi I., Mantica G., Minoli D.G., De Marco E.A., Gnech M., Bebi C., Manzoni G., Berrettini A. (2022). Fluorescence-Guided Surgery and Novel Innovative Technologies for Improved Visualization in Pediatric Urology. Int. J. Environ. Res. Public Health.

